# Precision Medicine in Rural Settings: Clinician Perspectives on Clinician-Ordered Genetic Testing

**DOI:** 10.21203/rs.3.rs-9851420/v1

**Published:** 2026-06-22

**Authors:** Anne C. Madeo, Kimberly A. Kaphingst, Melissa Yack, Erin D. Bouldin, Chelsey R. Schlechter, Sarah Dallas, Gwennndolyn Porter, Jennie L. Hill

**Affiliations:** a Department of Population Health Sciences, University of Utah, 295 Chipeta Way, Williams Building, Suite 16, University of Utah, Salt Lake City, UT, 84108, USA; b Huntsman Cancer Institute, University of Utah, 1950 Circle of Hope Dr., Salt Lake City, UT, 84112, USA; c Department of Communication, University of Utah, Languages and Communications Building, 255 S. Central Campus Dr., Room 2400, Salt Lake City, UT, 84112, USA; d Department of Internal Medicine, University of Utah School of Medicine, 30 N. Mario Capecchi Dr., 3^rd^ Floor North, Salt Lake City, UT, 84112 USA; e IDEAS Center, George E. Wahlen Department of Veterans Affairs Medical Center, 500 Foothill Boulevard, Salt Lake City, UT, 84148, USA; f Gwenn Porter Consulting, LLC PO Box 32, Boys Town, NE, 68010, USA

**Keywords:** Humans, Implementation Science, Precision Medicine, Rural Population, Health Personnel, Genetic Testing, Genetic Carrier Screening, Qualitative Research, Delivery of Health Care, Pharmacogenomic Testing

## Abstract

**BACKGROUND:**

Previous research has identified a limited number of specialty care providers (such as clinicians with specialty training in genetics) in rural areas. We consider here the perceptions of rural clinicians regarding genetic testing, as they may contribute to its incorporation into rural healthcare.

**METHODS:**

We conducted semi-structured interviews with rural clinicians in the Mountain West states of Utah, Montana, Nevada, and Wyoming who had discussed reproductive genetic carrier screening or disease risk genetic testing with a patient. Interviews were thematically analyzed using a hybrid deductive–inductive approach informed by the Practical, Robust Implementation and Sustainability Model (PRISM) and the Implementation Outcomes Framework (IOF), with a focus on contextual determinants and acceptability and appropriateness as implementation outcomes.

**RESULTS:**

We analyzed data from 19 rural clinicians who had discussed disease-risk genetic testing and/or reproductive genetic carrier screening with a patient. Participants generally perceived these discussions as acceptable and appropriate. The clinical actionability of information gleaned from genetic testing particularly influenced its acceptability. Clinicians expressed concern about selecting and interpreting genetic tests. Most clinicians did not describe working in a setting where genetic testing was a routinized part of the patient care workflow.

**CONCLUSIONS:**

Among Mountain West rural clinicians who discussed genetic testing with a patient, genetic testing was perceived as acceptable and appropriate, particularly when the results would affect patient management. However, rural clinicians were concerned about their knowledge base in selecting and interpreting genetic tests and relied on referrals to genetics specialists for these tasks. These findings suggest that implementing genetic testing in the rural Mountain West may require a coordinated effort to increase clinicians’ knowledge of genetic tests and their interpretation and ensure they have adequate access to support from genetics-trained colleagues.

## BACKGROUND

Ensuring that all U.S. residents experience the benefits of precision medicine, an approach to disease treatment and prevention that considers individual variability in genes, environment, and lifestyle [1], may require that genetic testing be accessible to a wider swath of the U.S. population. Currently, due to the traditional genetic testing service delivery model [2, 3] and the lack of genetics-trained specialists outside urban areas [4–6] and academic medical centers [7], obtaining genetic testing may be difficult in rural areas [8–10].

Despite this, genetic testing is a recommended component of decision-making related to cancer screening, prevention and treatment [11–14], heart disease treatment [15, 16], and reproductive decision-making [17–19]. Genetic testing that occurs as a part of cancer or heart disease treatment decisions and risk assessment, or disease risk genetic testing (DRGT), is recommended by multiple professional societies for individuals with a personal or family health history that is indicative of these adult-onset conditions [11, 13–15, 20, 21]. Similarly, professional society guidelines recommend that all pregnant individuals or those considering reproduction be offered reproductive carrier genetic screening (RGCS), ideally prior to initiating a pregnancy [17, 18, 22].

Rural clinicians may experience barriers to providing genetic services that are unique to their setting, including spatial distribution of healthcare facilities [23], difficulties in recruiting and retaining providers [24] and demographic trends of rural residents (e.g., rural residents are more likely to be older [25], less likely to have health insurance [26], and have lower levels of educational attainment [27]). Research that included clinician informants (many of whom do not practice in rural communities) indicates that they have been slow to adopt genetic screening due to perceptions of low self-efficacy [28], limited time [29], and limited access to services such as genetic counseling [30]. Clinician-related barriers to genetics *referral* in rural areas include limited knowledge of genetics and genetic conditions, limited awareness of genetic services, inadequate referral coordination, and a shortage of the genetic workforce [31–34].

Previous research has suggested that women living in rural areas may be more likely than those in suburban or urban areas to obtain genetic testing from a primary care provider [35] compared to a private genetic testing office/hospital. Supporting this finding, in our previous work interviewing 30 rural Mountain West residents who discussed DRGT or RGCS with a clinician [36], we found that access to this guideline-recommended genetic testing was patient-driven in the context of the limited local specialist availability that is characteristic of rural healthcare [37]. It is unknown whether rural clinicians in the U.S. feel prepared to discuss and offer genetic testing to their patients, their perceptions of its acceptability and appropriateness, and other factors that may contribute to the implementation of genetic testing in rural areas.

To better understand the contextual determinants of clinician-ordered genetic testing and perceptions of its acceptability and appropriateness among rural clinicians in the Mountain West who have discussed RGCS or DRGT with a patient, the research team interviewed rural Mountain West clinicians about their experiences discussing genetic testing with a patient and their perceptions of RGCS or DRGT acceptability and appropriateness.

## METHODS

We followed the COREQ (COnsolidated criteria for REporting Qualitative research) Checklist for reporting the study results [38].

### Ethics Review

This study was reviewed by the University of Utah Institutional Review Board (IRB_00176898) which deemed it exempt from documentation of consent. All participants received a consent cover letter, and prior to the interview, verbal informed consent was obtained. Participants affirmed their willingness to participate and have their interview recorded.

### Conceptual model

To guide the identification of key factors in the implementation of genetic testing, this study was informed by a theoretical model that integrated the Practical, Robust Implementation and Sustainability Model (PRISM [39]) and the Implementation Outcomes Framework (IOF [40]) (Figure 1). PRISM was selected for its pragmatic focus on understanding the contextual domains that affect the effectiveness of healthcare interventions in real-world settings. It includes the following multilevel contextual domains: (1) Intervention: Clinician/implementer perspectives, (2) Characteristics: Clinician/implementer, (3) External Environment, and (4) Implementation and Sustainability Infrastructure [41].

For this study, clinician perspectives on genetic testing included the strength of evidence for genetic testing, its value and barriers and facilitators to offering it in the clinician’s current clinic. Clinicians could also share their perceptions of others’ perspectives on genetic testing (e.g., patients, other clinicians, the organization). Clinician characteristics included their skills, capacity, and sociodemographic characteristics. Like perspectives on interventions, characteristics were measured at multiple levels (i.e., patient, clinician, organization). Beyond demographic characteristics, characteristics are also factors that will influence the fit between an individual or organization, like health literacy, and an intervention, like genetic testing. The External Environment included rurality (including patient travel time to appointments), policy and regulatory demands, community resources (e.g., distance to healthcare facilities and specialists), and market forces (e.g., testing lab contracts with insurers). The Implementation and Sustainability Infrastructure was defined to include the clinic’s resources and capacity to implement genetic testing, such as whether the clinician’s clinic currently offers genetic testing, staff roles and responsibilities, and clinic characteristics such as clinician training and resources (e.g. EHR capabilities, internet access).

IOF was selected for its focus on implementation outcomes that are crucial for understanding implementation effectiveness and the suggestion that implementation outcomes could be a target for implementation strategies [42]. In this study, we considered the IOF implementation outcomes of acceptability (“the perception among implementation stakeholders that a given treatment, service, practice, or innovation is agreeable, palatable, or satisfactory” [40]) and appropriateness (“the perceived fit, relevance, or compatibility of the innovation or evidence based practice for a given practice setting, provider, or consumer; and/or perceived fit of the innovation to address a particular issue or problem” [40]), as they are pre-implementation (e.g. implementation development or planning) outcomes that can be measured with semi-structured interviews at the individual level [40].

### Study sample

Participants were recruited with a purposive sampling strategy from multiple sources including emails about the study to rural clinic practice managers, Primary Health Care Associations and Offices of Rural Health in Idaho, Montana, Nevada, Wyoming and Utah, Wyoming and Montana State Medical Societies, Montana and Nevada State Oncology Societies, Montana and Idaho Cancer Control Programs, Utah Breast and Cervical Cancer Program, Utah Cancer Coalition, and Montana Obstetric and Maternal Support Program. These emails included a recruitment flyer for distribution to rural clinicians. In addition, we sent emails directly to licensees of the Montana Medical Examiners Board, the Nevada State Board of Nursing, the Utah Division of Professional Licensing who lived in rural counties, and to clinicians at the Central Utah Public Health Department.

### Sample size

Our *a priori* minimum sample size was 15 individuals, based on typical sample sizes that are sufficient to achieve thematic saturation [43–46] and we completed 19 interviews.

### Eligibility

The eligibility criteria for this study included being a practicing clinician who provided patient care in a rural county (defined by 2023 Rural Urban Continuum (RUC) [47] codes 4 – 9) in one of five Mountain West states (Idaho, Montana, Nevada, Wyoming, and Utah), having discussed DRGT or RCGS with a patient, and feeling comfortable participating in a conversation conducted entirely in English using a video conference service (e.g., Zoom ^®^). Individuals could present for the interview alone or accompanied by whomever they wanted, from any location.

### Screening potential participants

Potential participants who had not previously contacted an investigator received ≤5 contacts, initially via email or text, inviting them to complete a REDCap (Research Electronic Data Capture) eligibility survey, hosted at the University of Utah [48–50], and providing a link to a consent cover letter. Individuals who completed the REDCap eligibility survey were informed whether a preliminary assessment indicated their eligibility. If eligible, they were invited to schedule a 30-minute interview via Zoom ^®^ (Figure 2).

Given the broad public-facing recruitment strategy and based on published recommendations [51, 52], a two-step screening process was used to reduce the likelihood of participation by people outside the geographic area of interest or other ineligible participants. Individuals who could not answer at least five screening questions concordant with their responses to questions in the REDCap eligibility survey were informed that the research team would follow up with them if additional participants were needed but interview questions were not pursued. Individuals whose responses to the second screen were concordant with their REDCap eligibility survey were interviewed. ACM and JLH discussed the eligibility of participants who were interviewed but were suspected of being ineligible (e.g. individuals who declined invitations to turn on their camera, whose sound quality/connection to the interview was poor, and/or who were unable to accurately report a recruitment venue with which the research team was familiar [51, 52]) and discarded interview data from individuals determined to be likely ineligible. Compensation of $75 was offered to all participants who completed the interview, although some declined.

### Interviews

The authors developed a semi-structured interview guide with core questions and optional follow-up probes (Supplementary Materials). The interview guide drew on the PRISM and IOF models as well as our previous research [53] and was designed to elicit respondent feedback across PRISM domains and IOF implementation outcomes. The guide was not pilot tested. Interviews were conducted over three months between April and June 2025 by ACM, a cisgender White woman doctoral student and board-certified genetic counselor who lives in an urban area of a Mountain West state that consists primarily of rural counties. She completed coursework and training in qualitative methods and semi-structured interviewing. She had no prior relationship with any participants. Although the consent cover letter described the study goals and background, and ACM answered participants’ questions about her interest in the topic, the interview guide did not formally address the interviewer’s goals and background. The interviewer took notes during and/or after interviews. No participants were interviewed more than once.

Interviews were conducted via Zoom ^®^ [54], audio and video recorded and ranged in length from 18 to 46 minutes (mean 28 minutes). The interviewer was alone in a private location in her home throughout all interviews. Audio recordings were professionally transcribed and deidentified.

Recruitment continued until we had interviewed 19 individuals (Figure 2). During analysis (which occurred September 2025 – February 2026), we noted that this sample size was sufficient to reach saturation, or the point at which no new codes emerged across consecutive interviews [45, 55]. We assessed code saturation by tracking the first occurrence of each unique code across interviews ordered chronologically and calculating the number of newly identified codes per interview [46, 56].

#### Analysis

We conducted a thematic analysis using a hybrid coding approach consistent with Boyatzis [57] and Crabtree and Miller [58]. We began with deductive codes based on an *a priori* codebook informed by the conceptual model, and added inductive codes to capture themes that we identified in the data and in a companion study [36]. The codebook and coding protocol were based on the companion study investigating the experiences of rural residents discussing clinician-ordered genetic testing with a clinician and described in detail elsewhere [36]. The codebook was not revised for interviews with clinicians; the initial development of inductive codes included review of the transcript from an interview with a clinician.

In September 2025, two trained research assistants (RAs), a senior qualitative researcher (JLH), and the interviewer (ACM) used Microsoft Excel [59] to manage and apply the initial deductive codebook to a transcript and identify emergent codes. Emergent codes were defined based on our initial coding of two transcripts from interviews with rural residents [36] and one interview with a rural clinician. After defining emergent codes, they were added to the codebook. Transcripts were subsequently independently coded by two RAs according to the finalized codebook (Supplementary material). ACM reviewed and reconciled the coding of transcript segments. Coding discrepancies that could not be addressed without input from RAs were reconciled based on their feedback. Participants were asked neither to provide feedback on the findings nor on the transcripts.

During analysis, we included coded segments from a participant who identified as both a rural resident and a rural clinician and whose interview analysis was reported in a previous study [36]. The segments included in this analysis were clearly framed from a clinician perspective (e.g., “I took care of many patients who…”, “The hard part is trying to help patients understand…”).

Themes were identified by sorting and examining text segments with shared codes, making connections among them; they were then reviewed by the analytic team [58]. This was an iterative process of immersion, crystallization/connecting, and corroboration [60] undertaken by ACM, JLH, GP, and SD. Dominant themes were defined as patterned meanings that recurred across the dataset and were central to addressing our research questions. Subthemes also contributed to the research question but were less prevalent, less fully developed, or more narrowly bounded than dominant themes; subthemes sometimes functioned as a specific aspect of a broader thematic pattern [61]. The research team reviewed and discussed summary tables with illustrative quotes. Rigor was supported throughout coding and analysis by researchers who recorded analytic memos and stayed close to the data.

## RESULTS

Data from 19 individuals who completed an interview were included in this study. Characteristics of participants are presented in Table 1. When available, the characteristics of the participant interviewed as a rural resident and a rural clinician are included in Table 1. Participants were primarily Advanced Practice Providers [62], with a mean age of 45 years (SD: 10.0) whose patients were primarily non-Hispanic White. Tables 2–6 summarize the dominant themes and subthemes identified for each of the PRISM constructs and the measured IOF outcomes, with representative quotes.

The following sections and supporting thematic tables summarize context-aligned PRISM themes and emergent themes. PRISM perspectives on an intervention can describe multiple levels (i.e., patients, clinicians, organizations) and may include factors such as the intervention’s patient-centeredness, barriers and facilitators to its use, service and access, its compatibility with existing workflows, its estimated impact, and ease of use [63].

### PRISM Context Domain: Perspectives on genetic testing

In this study, clinicians’ perspectives on genetic testing centered on its clinical actionability, and on its impact (emotional and cost) on their patients. Their perspective included uncertainty about how to order and interpret genetic tests. Clinicians’ persectives on patients’ perceptions of genetic testing focused on patients’ concerns and motivations for genetic testing. Only three clinicians commented on organization perspectives on genetic testing, which they generally considered to be supportive or neutral.

#### Clinicians’ perspectives

Themes from participants’ perspectives on genetic testing (Table 2) indicate that clinicians perceived genetic testing as valuable when the results are clinically actionable (i.e., inform their management of a patient). For example, one clinician noted that a positive genetic test result would change when they recommended initiating cancer screening (C-27), while another clinician emphasized that genetic therapies for spinal muscular atrophy (SMA) give testing clear “utility” beyond reassurance (R-03). Similarly, a clinician described pharmacogenetic testing as helpful when it allowed the clinician to “guide the treatment and stop throwing a dart” after failed trials of medications (C-31). Across DRGT and RGCS, clinical actionability was central to positive perceptions of genetic testing.

At the same time, some clinicians indicated that uncertain or distressing results may do more harm than good. Several clinicians expressed concerns about the potential patient psychological burden of uncertain or distressing genetic test results, their ability to select (“I’m not sure what the right test is to order” (C-24)) and interpret genetic tests, and structural/social constraints (i.e. cost and insurance coverage) that reduce patient access to genetic testing. Cost uncertainty complicated their decisions. For example, clinicians worried about exposing patients to unexpected costs and encouraged patients to check whether their insurance would cover testing before ordering it. Clinician perspectives reflect a balance between recognizing the clinical utility of genetic testing and its role in patient management and concerns about its limitations in practice.

#### Clinicians’ perceptions of patients’ perspectives

Themes from clinicians’ perceptions of patients’ perspectives on genetic testing (Table 2) suggest that clinicians understand patient interest in genetic testing as influenced by family history and concern for their children, their limited knowledge about genetic testing, reluctance to receive potentially distressing information, and cost and insurance considerations. A subtheme of family history and concern for their children was that some patients pursue genetic testing in search of clarity or answers (‘I just wanna know.’ (C-12)). Despite patients’ interest in genetic testing, it was frequently “not on [patients’] radar” (C-09) and patients often had a limited understanding of genetic testing. Clinicians repeatedly identified genetic testing cost and insurance as central considerations in patient decision-making, noting that concerns about coverage, high deductibles, and potential bills could discourage patients from moving forward with testing. Clinicians also described patients being reluctant to receive information, particularly information that might be distressing or anxiety-provoking.

#### Clinicians’ perceptions of organization perspectives

Few clinicians commented on their organizations’ perspectives on genetic testing; those that did (n=3) indicated that they perceived their organizations as neutral towards or supportive of genetic testing (Table 2).

### PRISM Context Domain: Characteristics

The dominant theme that arose in clinicians descriptions of their characterics was a strong affinity for rural healthcare practice. When describing patient characteristics, their comments were most often about patient barriers patient barriers to accessing genetic testing. The dominant themes in organization characteristics were related to how clinicians within an organization identify and refer patients for genetic testing.

#### Clinician characteristics

Clinicians described a strong personal and professional identification with rural healthcare practice (Table 3), best summarized by the statement, “I just like rural medicine.” (C-24) Participants described enjoying the lifestyle of rural communities, valuing the breadth of their practice, and maintaining close relationships with their patients.

#### Patient characteristics

Clinicians who described patient-level characteristics that affect genetic testing (Table 3) consistently identified two types of patient characteristics that acted as barriers: financial and non-financial barriers to access. Financial barriers included difficulty affording fuel/gas, concerns about unexpected medical bills, and limited socioeconomic resources. Non-financial barriers included medical mistrust, digital access, and a refusal or inability to travel long distances to metropolitan areas. These characteristics were perceived as limiting patients’ access to genetic testing.

#### Organization characteristics

Clinicians indicated that organizational characteristics strongly influenced whether and how genetic testing was discussed (Table 3). Frequently, clinicians relied on referral-based care models for patients to discuss genetic testing with an outside specialist or genetic counselor, despite recognizing that patients may not be able or willing to travel for care. Although clinicians discussed offering healthcare via telehealth, only one clinician described telehealth as making genetic services more accessible to patients by making outside specialists more available. Organizations’ referral-dependent infrastructure placed genetic testing as an external service rather than an integrated part of routine care. Further limiting access to genetic testing, many organizations did not have standardized protocols to identify eligible patients and consistently offer genetic testing. Several clinicians reported that there wasn’t a “clear protocol” regarding genetic testing, they didn’t have guidance on when or how to discuss genetic testing, and they relied on changing laboratories to order genetic testing and services for genetics referral. This lack of standardization contributed to variability in practice.

### PRISM Context Domain: External Environment

The external environment is outside the clinicians’ organization. Themes that arose included factors such as insurance coverage and authorization policies, the challenges of a rural environment and other factors that limited uptake of genetic testing (e.g., knowledge, trust and healthcare system complexity).

External environmental factors were consistently described as structural determinants of access to, and uptake of, genetic testing (Table 4). Insurance coverage and authorization policies were barriers to genetic testing uptake. Clinicians reported that certain genetic tests required prior authorization and described uncertainty about whether authorization would be granted. Beyond insurance, clinicians emphasized that the absence of specialists in their communities limited local genetic testing capacity.

Even when genetics referral pathways were available to clinicians, rural patient follow-through was often limited by travel and transportation barriers. When patients had to travel to distant cities for testing weather could delay specialty visits, and unreliable transportation reduced the feasibility of a referral-based model [36]. In addition to these constraints, participants described community-level factors as influencing uptake. A clinician indicated that in some cultures, the term “genetic testing” was perceived as threatening. Another clinician reported that patients were unaware genetic testing was available and the overall complexity of the healthcare system limited uptake of genetic testing. While these sociocultural factors were less structural than insurance or geographic barriers, they were nonetheless aspects of the external environment in Mountain West rural clinics that influenced whether rural patients ultimately pursued genetic testing.

### PRISM Context Domain: Implementation and Sustainability Infrastructure

Themes that arose in the implementation and sustainability infrastructure included the reliance of organizations on external systems to provide genetic testing and gaps in workforce, clinician roles and administrative support.

Participants described interrelated organizational conditions that limited genetic testing implementation and sustainability (Table 5). First, workforce limitations and role strain (e.g. current insurance payment model and insufficient staffing) reduced clinicians’ ability to provide genetic testing. Administrative and organizational gaps further undermined the sustainability of genetic testing. One participant stated, “I just didn’t have any support from an administrative role.” (C-12) In their current clinical settings, participants indicated that genetic testing relied on external systems (“A lot of our testing has to be sent out.” (C-11)) and locally coordinated workflows (“We’re the middle man in it all. We’re the ones that end up, sometimes, having to try to figure things out…” (C-21)) Participants described these limitations as occurring within a reimbursement environment in which clinicians are “paid for patient-facing hours,” making time spent coordinating testing, communicating with specialists, or managing results uncompensated and undermining the sustainability of current systems. The combination of workforce limitations, administrative gaps, and reimbursement structures that prioritize billable encounters were described as creating challenges for integrating genetic testing into routine care.

### Implementation Outcomes Framework

#### Acceptability

Genetic testing acceptability was influenced by patients’ responses to genetic testing, its perceived clinical value and interpretability.

Clinicians’ acceptability of genetic testing was strongly linked to its perceived clinical actionability. (Table 6) When genetic test results might inform clinician actions caring for patients, such as medication selection, preventive screening, or disease prevention, clinicians described it as valuable. In contrast, when clinicians perceive genetic testing interpretability as low, its acceptability was also low. Clinicians expressed concerns about their abilities to interpret genetic tests. Clinicians also reported on the acceptability of genetic testing to their patients, indicating that patient acceptability was contingent on patients’ emotional responses to and beliefs about genetic testing.

#### Appropriateness

Genetic testing appropriateness is defined as its “perceived fit, relevance or compatibility for a given practice setting,” clinician, or patient and/or its “perceived fit to address a particular issue or problem” [40]. It is characterized by descriptions of the suitability of genetic testing, its relevance to personal or clinical needs, and reflections on its timing, setting or population fit [40]. Clinicians described the appropriateness of genetic testing (Table 6) as influenced by their perceptions of its clinical relevance, compatibility with their role and clinical setting, and their patient’s resources. Genetic testing was perceived as appropriate when it clearly guided patient care. Appropriateness was diminished in settings where clinicians did not provide longitudinal follow-up (e.g., emergency and radiology departments) or where testing was perceived as outside the scope of the indication for care. Further, clinicians described the appropriateness of genetic testing as potentially reduced or facilitated by contextual issues. In short, clinicians’ perceptions of appropriateness were influenced not only by clinical utility but also by the perceived fit of genetic testing within their roles, settings, and areas of expertise.

## DISCUSSION

In this qualitative study of rural clinicians in the Mountain West who had discussed RGCS or DRGT with a patient, we identified contextual factors that influence how clinicians discuss and access genetic testing for patients. Guided by PRISM [39] and IOF [40], our findings suggest that genetic testing discussed with a patient was generally perceived by clinicians as acceptable and appropriate, particularly when the results would affect a patient’s medical management (i.e., facilitate medication selection, identification of preventive therapies/surgeries, change screening recommendations). However, clinician perceptions of acceptability and appropriateness were conditional on clinician understanding of a patient’s clinical scenario, the interpretability of genetic test results, and their clinical setting.

Previous research suggests that clinician uncertainty about out-of-pocket patient costs and insurance coverage of genetic testing (Table 2) is well-founded. In the U.S., some genetic tests are not widely covered by insurance (e.g. panel tests), leaving patients to pay out of pocket costs and clinicians to negotiate prior authorization requirements [64]. A recent evaluation of medical policy determinations found significant variability in policy determinations [65]. Based on previous research, concerns about genetic testing insurance coverage and cost are unlikely to be limited to rural areas [64, 66].

The current study aimed to gather rural clinician perspectives on genetic testing in clinical care and found that these perspectives are similar to those of non-rural clinicians reported by others. Barriers that were not discussed by rural clinicians in this study that have previously been described by clinicians include lack of time to incorporate conversations about family history [29] and genetic testing, lack of genetic workforce in rural areas [31, 32] and concerns about the utility of pharmacogenomic testing [67]. Although rural clinicians in this study did not specifically comment on the availability of specialists with genetics training in rural areas, this may be due to the broad lack of specialists in rural areas [37], which they did comment on. Rural Mountain West residents described inadequate referral coordination as a barrier to obtaining genetic services [36], but the referral barrier most frequently noted by clinicians was that patients often would not or could not present for referrals to specialty visits in distant cities. This difference suggests that our previous research with rural residents may not have included participants who were unwilling or unable to be seen at remote specialty clinics.

This research suggests that clinicians conditionally believe that genetic testing is appropriate for their patients. Specifically, genetic testing is appropriate for patients who are perceived to be at high risk to harbor a pathogenic variant and for whom genetic test results would be expected to impact clinical management and when the clinician is in a specialty for which genetic testing is perceived to be appropriate.

In rural contexts, where access to specialty services is limited [37], non-genetics trained clinicians may play a particularly important role in influencing patients’ understanding and decision-making regarding genetic testing. Previous research with rural residents who obtained BRCA testing [35] and clinicians’ personal and professional identification with rural healthcare (Table 3) suggests that rural clinicians may be well positioned to serve as primary initiators of genetic testing conversations with patients. Rural workforce literature highlights the generalist orientation and expanded role expectations common in rural practice [24]. The limited availability of specialists and clinicians’ identification with rural healthcare might support a model in which genetic testing is “mainstreamed” (non-genetics-trained clinicians provide alternative pre-test counseling, such as standardized educational materials, order genetic testing and then genetic counselors provide post-test counseling [68, 69]). As individuals who embrace a breadth of practice opportunities, rural clinicians may be uniquely willing to learn about, discuss and offer genetic testing to patients, although this task may pose an additional burden on them. The barriers they presented to these actions could be addressed through Expert Recommendation for Implementing Change (ERIC) implementation strategies that include conducting “educational outreach visits”, developing “academic partnerships,” “making billing easier”, placing genetic testing on fee for service lists, and using “other payment schemes.” [70]. Acknowledging rural clinicians’ autonomy and generalist expertise in the context of these strategies may be particularly effective. The clinicians interviewed in this study were primarily Advanced Practice Providers [62], who may have a strong preference for a cohesive interprofessional team [71, 72]. This preference may further support a mainstreaming model of genetic testing, in which Advanced Practice Providers may work with an interprofessional team.

These findings help contextualize prior quantitative analyses, such as our own work using nationally representative survey data, which found no rural–urban differences in the prevalence of genetic testing [53, 73]. The present study suggests that similar genetic testing prevalence does not imply similar means to obtaining genetic testing. Rural clinicians described adaptive strategies—such as a reliance on referrals—to enable testing despite structural constraints. A scoping review of genetic service delivery models similarly demonstrates heterogeneity in operational approaches across practice settings [74].

These findings have implications for the delivery of genetic services in rural regions. Previous researchers have identified barriers to implementation of primary care risk assessment and offer of genetic testing that occur at the patient [75], clinician [76, 77], and organization [74] levels, and the external environment [39] [78] in non-rural settings, yet risk assessment and genetic testing may happen in the primary care setting [79]. Our research has identified barriers at the patient, clinician, and organizational levels, within the External Environment, and in the Implementation and Sustainability Infrastructure. Despite these barriers, there are facilitators, including rural clinician characteristics, organization perspectives on genetic testing and the acceptability and appropriateness of genetic testing to clinicians and rural residents [36]. Efforts to improve patient access to genetic testing in rural clinics may be facilitated by mainstreaming genetic testing so that it is integrated into rural clinicians’ routine workflows, guided by evidence and clinic policy, and supported by specialists [80, 81].

This study has limitations. Clinicians interviewed had already discussed genetic testing with a patient; therefore, our findings do not capture barriers experienced by clinicians who have not discussed genetic testing with a patient (e.g., clinicians who are unaware of genetic testing or practice in settings or specialty services where it is not routinely discussed). The majority of clinicians in this study discussed DRGT with a patient (Table 1), although we attempted to recruit clinicians who had discussed RGCS with a patient. This limits our ability to describe the perspectives and experiences of rural clinicians who discussed RGCS with a patient. Future research might sample exclusively from specialty services that already integrate RGCS into their practice (i.e., OB/GYN) to recruit more individuals who discussed RGCS with a patient. The recommendations for implementation of RGCS (which enjoys greater insurance coverage when not offered as an expanded panel [82, 83] may be different than implementation of DRGT. The sample was limited to English-speaking clinicians in the Mountain West, which may limit generalizability to other rural regions (particularly those that do not share the Mountain West’s geographic [84, 85] and social features [86]) or to non-English-speaking populations.

## CONCLUSION

Mountain West rural clinicians who discussed clinician-ordered genetic testing with patients generally viewed it as acceptable and appropriate, but identified multiple barriers to offering it themselves. Barriers were identified across all PRISM domains and at multiple levels. Clinicians perceived genetic testing insurance coverage barriers for themselves and patients, as well as cost barriers for their patients. Similarly, clinicians indicated that insurance coverage and authorization policies were an external environment barrier to patients’ access to genetic testing. Therefore, identifying implementation strategies that can address these barriers across levels/domains may be impactful areas for rural genetic testing implementation.

## Supplementary Material

Supplementary Files

This is a list of supplementary files associated with this preprint. Click to download.

- interviewGuideClinicianv4.docx
- Codebook.xlsx

## Figures and Tables

**Figure 1.** Conceptual model that informed study. Based on the Practical, Robust Implementation and Sustainability Model [39] and the Implementation Outcomes Framework [40].

**Figure 2.** Participant selection.

**Table 1.** Participant characteristics

	N = 19
**Age (years) (M, range, SD)**	45, 29 – 71, 10.0
	
**Clinician type**	N = 19
Nurse	1
Nurse Practitioner	8
Physician Assistant	5
Physician	5
	
**Race/Ethnicity of majority of clinicians’ patients**	N = 19
**Hispanic/Latino/Latinx**?	0
**Race or origin** (categories not mutually exclusive)	
White	18
American Indian or Alaska Native	4
**County RUC**^a^ [47] **code of facility where clinician sees patients**	N = 18
4^a^	2
5^a^	2
6^a^	3
7^a^	5
8^a^	1
9^a^	5
	
**State where clinic located**	N = 19
Idaho	0
Montana	5
Nevada	4
Utah	9
Wyoming	1
	N = 18
**Time Practicing in Rural Community (years)** (M, range, SD)	7, (1 – 20), 12.7
	
**Type of genetic testing discussed with a patient**	N = 19
Reproductive carrier genetic screening	3
Disease risk genetic testing	16

a RUC code county descriptions: 4 = Urban population of 20,000 or more, adjacent to a metro area; 5 = Urban population of 20,000 or more, not adjacent to a metro area; 6 = Urban population of 5,000 to 20,000, adjacent to a metro area; 7 = Urban population of 5,000 to 20,000, not adjacent to a metro area; 8 = Urban population of fewer than 5,000 adjacent to a metro area; 9 = Urban population of fewer than 5,000 not adjacent to a metro area

**Table 2.** Themes and illustrative quotes from PRISM contextual domain of perspectives on genetic testing.

Clinicians’ Perspectives on Genetic Testing	
Themes	Quotes
Importance of clinical actionability	“Maybe [genetic testing] would be helpful… to guide the treatment and stop throwing a dart.” (C-31)
	“When they have the genetic therapy for SMA now… it gives a utility to the test instead of just, oh, everything’s perfect.” (R-03)
	“If there’s something you can do preventatively… knowledge is power… get on earlier testing, early colonoscopies or early mammograms.” (C-27)
Uncertain or distressing results may do more harm than good	“If we were to order those… and they were to come back positive… then they’re like, ‘Well, what do I do with this?’… it induces maybe some anxiety.” (C-08)
	“Maybe it’s possible that they may be better off just not knowing… a lotta times… the information that we give patients can just increase anxiety.” (C-11)
	“The other thing is it feels sometimes—as a clinician it can very much feel like a scare tactic of this really horrible thing could happen. You’re like I’m not looking to scare people into something.” (R-03)
Uncertainty about ordering and interpreting genetic tests	“I’m not sure what the right test is to order…I know the basic ones…but whenever I’ve sent a patient to a geneticist… they always order more advanced labs than I would have thought of.” (C-24)
	“Then if I dont’ [know how to manage it], then I’m like, ‘Hey, this is out of my wheelhouse. I wanna make sure I’m not misinterpreting this for you, so I really think we should send you to a geneticist to be able to talk about these results.’” (C-16)
	“You don’t want to order the wrong test.” (C-20)
Concerned about cost and coverage of genetic testing	“I usually put the patient in charge… they can call their insurance and check first.” (C-22)
	“I’m not even sure… if it’s not covered, what it would cost. I guess I should probably know that as a provider, but I don’t.” (C-28)
	“If they don’t qualify… knowing then that insurance wouldn’t pay for it, it would be out of pocket… and then let them decide if they want to test or not.” (C-25)
Clinicians’ perceptions of patient perspectives on genetic testing	
Family history and concern for children increases interest	“Also, a young [patient] with I want to say [cancer type]… [They] did have children and wanted to know if that was something [they] needed to worry about with [them]. (C-27)
	“…the patient had come in and specifically requested [genetic] testing because of family history.” (C-22)
	“[Patient] had kids, and so this was something that just—do I have something that I could give to my children that I need to worry about now to have them tested for in the future?” (C-32)
Themes and Subtheme	Quotes
**Subtheme:** Desire for clarity or answers motivates genetic testing	“The patient wanted to make sure that [they] either did or didnt’ have it. I guess, how that would affect [their] life.” (C-08)
	“[Patient] didnt’ have any features. [They were] like, ‘Do [genetic testing]. That’s fine. That sounds good.’ [They’re] like, ‘I just wanna know.’” (C-12)
	“I have had a couple bring [genetic testing] up themselves. Again, it was patients who had a longer, complicated history, had been through treatment for a while, and they said, ‘We’ve heard, or we read about this, and maybe it would help my case.’” (C-31)
Reluctance to know	“I just think that [people in their 80s and 90s]’re just not interested [in genetic testing]. They dont’ really see a real benefit, even if you tell ‘em ‘bout the kids…” (C-09)
	“[Some patients]… say, ‘I’d rather not know.’” (C-27)
	“I guess, in a certain sense, sometimes, no because it can induce more anxiety or questions. When we don’t have the answers either, then there’s just that uncertainty there where it’s like, ‘Okay, well, but how is this gonna affect me and why does nobody know the answer?’ kind of thing.”“ (C-08)
Limited knowledge/understanding	“It’s just lack of knowledge. The internet’s out there, and it’s a bit of a mess.” (C-09)
	“I didn’t really pay attention to what [direct-to-consumer genetic test] tests for, but I know people do bring it up and say, ‘Yeah, I’m not so worried about things because I had a saliva test done.’” (C-28)
	“I just think it’s just the lack of understanding [among patients] of [genetic testing] and just not having the education with it being out here…” (C-21)
Cost and coverage concerns	“Most of our patients have high deductible plans. Most of them have a health savings account but in the case of a [patient] whose [sibling] had been diagnosed with [organ] cancer, [they] had said, ‘I have money in my HSA, I want to do the test, I’m not worried about the cost.’” (C-22)
	“…there’s also a big concern. It’s a smaller community, also lower socioeconomic, and so cost and insurance coverage is always top.” (C-25)
	“Sometimes it’s…because insurance wont’ pay for [genetic testing].” (C-18)
Clinician understanding of organization perspectives on genetic testing	
Theme	Quotes
Organizations are supportive or neutral	“…most of the time…[the larger system]’s no issue, and it doesnt’ really play a role either pro or con…” (C-16)
	“I would say everyone in this clinic is very onboard for the [reproductive carrier genetic] testing.” (C-25)

**Table 3.** Themes and illustrative quotes from PRISM contextual domain of clinician, patient or organizational characteristics.

Clinician characteristics	
Themes	Quotes
Personal and professional identification with rural healthcare	“I just like rural medicine… Most of my patients I know on a professional level and on a personal level, just because we’re so small. I see my patients at the grocery store. I see them everywhere.” (C-24)
	“In my clinical year…I did a lot of serving the underserved… When I had the opportunity to move up here, I was like, ‘That’s something I wanna do. It fits perfectly.’” (C-08)
	“…it’s just the diversity of rural medicine. I get to do a lotta stuff, a lotta different things…”(C-21)
Clinician understanding of patient characteristics	
Themes	Quotes
Financial barriers to access	“Not everybody has the means to pay for gas.” (C-09)
	“I’ve even had patients be like, ‘I’m here for my physical, but only my physical.’ Like, ‘Please don’t do one test above, because last time, I got slapped with a huge bill this way.’” (C-20)
	“There’s a big, I would say, socioeconomic gap up here anyways. People that are able to, will go.” (C-08)
Non-financial barriers to access	“Some [rural residents] just refuse to go up to <City> an hour and a half away. They just dont’ wanna do it.” (C-09)
	“We do phone visits, but a lot of our patients here dont’ have Zoom, or some of ’em dont’ even have computers. That’s about the extent of it, is a phone visit, so.” (C-27)
	“There is some of that mistrust around medicine and stuff. That’s something you have to navigate a lot.” (C-08)
Clinician understanding of organization characteristics	
Themes	Quotes
Genetic testing managed primarily through referrals	“No [genetic services in our system]…we just fax [a referral to genetics] over to the [medical facility ~211 miles from clinic location] or <Hospital> or wherever the patient’s going to be seen.” (C-24)
	“One of the [clinicians]…just said that’s [that referals had to be made for genetic testing] how it always was.” (C-08)
	“We use <EHR name>…and I just refer to—for genetic testing.” (C-32)
Lack of standardized protocols	“There was no policy across the board on [pharmacogenetic testing] either….there just wasn’t an office-wide agreement on like, ‘When do we do, how do we do?’” (C-20)
	“There’s been a couple of programs over my time at the clinic… if I wanted or a patient wanted any genetic testing, I had to contact somebody… and they would recommend what testing and then I could order it.” (C-22)
	“…we dont’ have that clear protocol of who to discuss genetic testing.” (C-30)

**Table 4.** Themes and illustrative quotes from PRISM contextual domain of external environment.

Theme	Quotes
Insurance coverage and authorization policies constrain access to genetic testing	“If some of the more proprietary disease-specific genetic testing… those have to be authorized by insurance. I have to go through the insurance and make sure that it’s covered before I can even order it.” (C-16)
	“We’re always hitting our head against the wall as far as like coverage. Will they cover [genetic testing] type of a thing?” (C-14)
	“Sometimes [genetic testing] has to go through their insurance. There’s a paper that we might fill out. Not all the time but some of the time.” (C-22)
Limited specialty care availability in rural settings	“we cant’ get somebody into a neurologist… because there’s not a neurologist for 300 miles.” (C-16)
	“We dont’ have a urologist at this time. We just barely got a gastroenterologist back in town. It is pretty sparse here.” (C-28)
	“We don’t have a lotta specialists in this area… a rural clinic, we struggle with that more.” (C-25)
Geographic and transportation barriers limit access to referred care.	“…transport is an issue in itself.” (C-08)
	“Really significant healthcare needs and barriers to transportation… Yes, you can refer them, but they wont’ go or they…dont’ have reliable transportation.” (C-12)
	“Sometimes… patients getting down to specialty visits can be delayed because of weather conditions.” (C-21)
Community awareness, trust, and system complexity limit genetic testing uptake	“The word ‘genetic testing’ in some cultures feels very attacking… The trust barrier was so huge….” (C-12)
	“I don’t know if you’ve tried to navigate the healthcare system, but it’s pretty overwhelming…I’ve had a really hard time navigating.” (C-28)
	“…the lack of knowing that [genetic testing]’s available.” (C-21)

**Table 5.** Themes and illustrative quotes from PRISM contextual domain implementation and sustainability infrastructure.

Theme	Quotes
Genetic testing currently relies on external systems and locally coordinated workflows	“We’re the middle man in it all. We’re the ones that end up, sometimes, having to try to figure things out…” (C-21)
	“A lot of our testing has to be sent out.” (C-11)
	“Usually, [genetic testing]’ll be put on a tracker that then goes to billing. Then they find out what pre-qualifications we need to meet to get it covered.” (C-16)
Workforce limitations and role strain constrain implementation	“The payment model of the current industry is…you’re paid for patient-facing hours. Me calling an endocrinologist and talking and doing these plans, all of that was my own free time.” (C-12)
	“We have two people that work on [genetic testing]. It’s not their full-time job, but it’s a major part of what they do.” (C-09)
	“We’d need more people…More providers. Yeah. We would need more of everything…Office staff and providers” (C-18)
Administrative and organizational gaps undermine sustainability	“I just didn’t have any support from an administrative role after multiple people had changed.…once it was built, I just couldn’t sustain it on my own.” (C-12)
	“As far as I understand, and that came down from administration…we havent’ pursued [telehealth].” (C-16)
	“That would be nice to have a policy… I’m not aware of what my colleagues do one way or the other.” (C-20)

**Table 6.** Themes and illustrative quotes from the Implementation Outcomes Framework (IOF) acceptability and appropriateness.

IOF: Acceptability	
Theme	Quotes
Perceived clinical value drives acceptability	“…if we’re using the [test name] for mental health, it’s a huge benefit because we can see what they’re going to metabolize and what medications we can use that way.” (C-30)
	“…[Genetic testing] could help inform maybe why the patient—things are going a certain way….it’s just a little bit more insight as far as care.” (C-19)
	“…[genetic testing]’s very important if there’s something you can do preventatively…knowledge is power…get on earlier testing, early colonoscopies or early mammograms…Prevention is key.” (C-27)
Perceived interpretability of genetic testing influences acceptability	“We dont’ really know how [a gene mutation] affects the body fully or what it means for our health in the future…Those kind of questions are difficult.” (C-08)
	“patients sometimes dont’ know what to do with it.” (C-11)
	“How do you explain that to somebody who doesnt’ even understand genetics… That’s really hard.” (R-03)
Patient emotional responses and beliefs influence genetic testing acceptability	“[Older patients] dont’ really see a real benefit…‘Whatever happens, happens. I dont’ care.’” (C-09)
	“I think [patient’s interest in genetic testing] was curiosity, but also some anxiousness…” (C-08)
	“There is a certain amount of stress or fear that can come from [genetic testing]…” (C-27)
IOF: Appropriateness	
Themes	Quotes
Clinical scenario and expected impact on care determine appropriateness	“They’ve had a blood clot…unusual circumstances…that might be an occasion where…they have a risk but they dont’ know it yet.” (C-22)
	“When I talk with my patients about ordering the metabolism testing…if they come in with a long history of having tried many medications…maybe this would be a case where that would be helpful, to get some more information to guide the treatment and stop throwing a dart.” (C-31)
	“…they have the genetic therapy for SMA now. It’s easy to be able to say, well, now it would helpful for you to know because then you could plan a consult…It gives a utility to the test instead of just, oh, everything’s perfect.” (R-03)
Provider clinical setting determines appropriateness	“In the emergency department, I dont’ think [genetic testing]’s affected anything that I’ve done.” (C-19)
	“I dont’ offer [genetic testing]… ‘cause I dont’ follow up on those results… I just play an auxiliary role and shuffle them to the right people…” (C-11)
	“Family practice, I think, is more appropriate” (C-24)
Contextual constraints (e.g. cost, access and patient resources) may limit appropriateness	“We’re always hitting our head against the wall…Will [insurance] cover it…” (C-14)
	“[Patient] wanted to have genetic testing done, but couldnt’ afford…$15,000…” (C-32)
	“Not everybody has the means to pay for gas.” (C-09)

## Data Availability

The datasets generated and analyzed during the current study are not publicly available due to concerns regarding the privacy of individuals who participated in the study but are available from the corresponding author on reasonable request.

## Abbreviations

COREQ: COnsolidated criteria for REporting Qualitative research
DRGT: Disease Risk Genetic Testing
IOF: Implementation Outcomes Framework
PRISM: Practical, Robust Implementation and Sustainability Model
RA: Research Assistant
RGCS: Reproductive Genetic Carrier Screening
RUC: Rural-Urban Continuum

## References

[1] What is precision medicine? [https://medlineplus.gov/genetics/understanding/precisionmedicine/definition/]

[2] Stoll K, Kubendran S, Cohen SA. The past, present and future of service delivery in genetic counseling: Keeping up in the era of precision medicine. Am J Med Genet C Semin Med Genet 2018, 178(1):24–37. 29512888 10.1002/ajmg.c.31602

[3] IOM (Institute of Medicine). In: Innovations in Service Delivery in the Age of Genomics: Workshop Summary. edn. Washington (DC): National Academies Press; 2009. 20662122

[4] Jenkins BD, Fischer CG, Polito CA, Maiese DR, Keehn AS, Lyon M The 2019 US medical genetics workforce: a focus on clinical genetics. Genet Med 2021, 23(8):1458–1464. 33941882 10.1038/s41436-021-01162-5 PMC8091643

[5] Villegas C, Haga SB. Access to Genetic Counselors in the Southern United States. In: Journal of Personalized Medicine. vol. 9; 2019. 10.3390/jpm9030033 PMC6789777 31266141

[6] Triebold M, Skov K, Erickson L, Olimb S, Puumala S, Wallace I Geographical analysis of the distribution of certified genetic counselors in the United States. J Genet Couns 2021, 30(2):448–456. 32929835 10.1002/jgc4.1331

[7] National Society of Genetic Counselors. Executive Summary, 2024 Professional Status Survey. In: 2024 Professional Status Survey. 2024.

[8] Shi L, Kolor K, Chen Z, Lu CY, Rodriguez J, Khoury MJ BRCA genetic testing utilization and expenditures among privately insured adults in the United States, 2013 to 2022. Genet Med 2025, 27(11):101556. 40847711 10.1016/j.gim.2025.101556 PMC13389943

[9] Gilbert KM, McLaughlin HM, Farmer JR, Ong MS. Disparities in Genetic Testing for Inborn Errors of Immunity. J Allergy Clin Immunol Pract 2025, 13(2):388–395 e383. 39579980 10.1016/j.jaip.2024.11.011 PMC11807750

[10] Curtin M, Somayaji D, Dickerson SS. Precision Medicine Testing and Disparities in Health Care for Individuals With Non-Small Cell Lung Cancer: A Narrative Review. Oncol Nurs Forum 2022, 49(3):257–272. 35446830 10.1188/22.ONF.257-272

[11] Bedrosian I, Somerfield MR, Achatz MI, Boughey JC, Curigliano G, Friedman S Germline Testing in Patients With Breast Cancer: ASCO-Society of Surgical Oncology Guideline. J Clin Oncol 2024:Jco2302225. 10.1200/JCO.23.02225 38175972

[12] Chen Y, Cao J, Huang R, Lou J, Gu J, Luo Z PARP Inhibitors in the Treatment of Prostate Cancer: An Analysis of the Clinical Trial Landscape. Cancer Med 2025, 14(21):e71298. 41165261 10.1002/cam4.71298 PMC12573472

[13] National Comprehensive Cancer Network. Genetic/Familial High-Risk Assessment: Colorectal, Endometrial, and Gastric. In: NCCN Clinical Practice Guidelines in Oncology. Plymouth Meeting, PA; 2026. 10.6004/jnccn.2024.0061 39689429

[14] National Comprehensive Cancer Network. Genetic/Familial High-Risk Assessment: Breast, Ovarian Pancreatic, and Prostate. In: NCCN Clinical Practice Guidelines in Oncology. Plymouth Meeting, PA; 2024. 10.6004/jnccn.2021.0001 33406487

[15] Sturm AC, Knowles JW, Gidding SS, Ahmad ZS, Ahmed CD, Ballantyne CM Clinical Genetic Testing for Familial Hypercholesterolemia: JACC Scientific Expert Panel. J Am Coll Cardiol 2018, 72(6):662–680. 30071997 10.1016/j.jacc.2018.05.044

[16] Gidding SS, Ballantyne CM, Cuchel M, de Ferranti S, Hudgins L, Jamison A It is Time to Screen for Homozygous Familial Hypercholesterolemia in the United States. Global Heart 2024. 10.5334/gh.1316 PMC11067975 38708402

[17] Committee Opinion No. 691: Carrier Screening for Genetic Conditions. Obstet Gynecol 2017, 129(3):e41–e55. 28225426 10.1097/AOG.0000000000001952

[18] Sagaser KG, Malinowski J, Westerfield L, Proffitt J, Hicks MA, Toler TL Expanded carrier screening for reproductive risk assessment: An evidence-based practice guideline from the National Society of Genetic Counselors. J Genet Couns 2023. 10.1002/jgc4.1676 36756860

[19] Gregg AR, Aarabi M, Klugman S, Leach NT, Bashford MT, Goldwaser T Screening for autosomal recessive and X-linked conditions during pregnancy and preconception: a practice resource of the American College of Medical Genetics and Genomics (ACMG). Genet Med 2021, 23(10):1793–1806. 34285390 10.1038/s41436-021-01203-z PMC8488021

[20] Hampel H, Bennett RL, Buchanan A, Pearlman R, Wiesner GL. A practice guideline from the American College of Medical Genetics and Genomics and the National Society of Genetic Counselors: referral indications for cancer predisposition assessment. Genet Med 2015, 17(1):70–87. 25394175 10.1038/gim.2014.147

[21] Syngal S, Brand RE, Church JM, Giardiello FM, Hampel HL, Burt RW. ACG clinical guideline: Genetic testing and management of hereditary gastrointestinal cancer syndromes. Am J Gastroenterol 2015, 110(2):223–262; quiz 263. 25645574 10.1038/ajg.2014.435 PMC4695986

[22] Grody WW, Thompson BH, Gregg AR, Bean LH, Monaghan KG, Schneider A ACMG position statement on prenatal/preconception expanded carrier screening. Genet Med 2013, 15(6):482–483. 23619275 10.1038/gim.2013.47

[23] Casey MM, Thiede Call K, Klingner JM. Are rural residents less likely to obtain recommended preventive healthcare services? Am J Prev Med 2001, 21(3):182–188. 11567838 10.1016/s0749-3797(01)00349-x

[24] Institute of Medicine. Quality Through Collaboration: The Future of Rural Health. Washington, DC, USA: The National Academies Press; 2005.

[25] Farrigan T, Genetin B, Sanders A, Pender J, Thomas KL, Winkler R Rural America at a glance: 2024 edition. In. Edited by U.S. Department of Agriculture Economic Research Service; 2024.

[26] Eisenstein S, Huang X, McBride T, Mueller K. Health Insurance Coverage in Rural and Urban Areas in the U.S., 2023. In.: RUPRI Center for Rural Health Policy Analysis.

[27] Employment & Education - Rural Education [https://ers.usda.gov/topics/rural-economy-population/employment-education/rural-education]

[28] Gramling R, Nash J, Siren K, Eaton C, Culpepper L. Family physician self-efficacy with screening for inherited cancer risk. Ann Fam Med 2004, 2(2):130–132. 15083852 10.1370/afm.60 PMC1466652

[29] Ahmed S, Hayward J, Ahmed M. Primary care professionals’ perceptions of using a short family history questionnaire. Fam Pract 2016, 33(6):704–708. 27535332 10.1093/fampra/cmw080

[30] Seibel E, Gunn G, Ali N, Jordan E, Kenneson A. Primary Care Providers’ Use of Genetic Services in the Southeast United States: Barriers, Facilitators, and Strategies. J Prim Care Community Health 2022, 13:21501319221134752. 10.1177/21501319221134752 PMC9647281 36345220

[31] Iredale R, Jones L, Gray J, Deaville J. ‘The edge effect’: an exploratory study of some factors affecting referrals to cancer genetic services in rural Wales. Health Place 2005, 11(3):197–204. 15774327 10.1016/j.healthplace.2004.06.005

[32] Koil CE, Everett JN, Hoechstetter L, Ricer RE, Huelsman KM. Differences in physician referral practices and attitudes regarding hereditary breast cancer by clinical practice location. Genet Med 2003, 5(5):364–369. 14501831 10.1097/01.gim.0000086477.00766.c9

[33] Butler R, Dwosh E, Beattie BL, Guimond C, Lombera S, Brief E Genetic counseling for early-onset familial Alzheimer disease in large Aboriginal kindred from a remote community in British Columbia: unique challenges and possible solutions. J Genet Couns 2011, 20(2):136–142. 20927575 10.1007/s10897-010-9334-9

[34] Harding B, Webber C, Rühland L, Dalgarno N, Armour C, Birtwhistle R Bridging the gap in genetics: a progressive model for primary to specialist care. BMC Med Educ 2019, 19(1):195. 31185964 10.1186/s12909-019-1622-y PMC6558677

[35] Dibble KE, Connor AE. Residential Locale Is Associated with Disparities in Genetic Testing-Related Outcomes Among BRCA1/2-Positive Women. J Racial Ethn Health Disparities 2023, 10(2):718–729. 35178668 10.1007/s40615-022-01259-w PMC8853067

[36] Madeo AC, Kaphingst KA, Yack M, Bouldin ED, Schlechter CR, Dallas S Precision Medicine in Rural Settings: Patient Perspectives on Clinician-Ordered Genetic Testing. Res Sq 2026.

[37] Cyr ME, Etchin AG, Guthrie BJ, Benneyan JC. Access to specialty healthcare in urban versus rural US populations: a systematic literature review. BMC Health Serv Res 2019, 19(1):974. 31852493 10.1186/s12913-019-4815-5 PMC6921587

[38] Tong A, Sainsbury P, Craig J. Consolidated criteria for reporting qualitative research (COREQ): a 32-item checklist for interviews and focus groups. Int J Qual Health Care 2007, 19(6):349–357. 17872937 10.1093/intqhc/mzm042

[39] Feldstein AC, Glasgow RE. A practical, robust implementation and sustainability model (PRISM) for integrating research findings into practice. Jt Comm J Qual Patient Saf 2008, 34(4):228–243. 18468362 10.1016/s1553-7250(08)34030-6

[40] Proctor E, Silmere H, Raghavan R, Hovmand P, Aarons G, Bunger A Outcomes for implementation research: conceptual distinctions, measurement challenges, and research agenda. Adm Policy Ment Health 2011, 38(2):65–76. 20957426 10.1007/s10488-010-0319-7 PMC3068522

[41] Frequently Asked Questions about PRISM [https://re-aim.org/learn/prism/]

[42] Morton K, Santillo M, Van Velthoven MH, Yardley L, Thomas M, Wang K Promoting the implementation of clinical decision support systems in primary care: A qualitative exploration of implementing a Fractional exhaled Nitric Oxide (FeNO)-guided decision support system in asthma consultations. PLoS One 2025, 20(2):e0317613. 39946372 10.1371/journal.pone.0317613 PMC11824951

[43] Morse JM. The Significance of Saturation. Qual Health Res 1995, 5(2):147–149.

[44] Miles MB, Huberman AM, Saldaña J. Fundamentals of Qualitative Data Analysis. In: Qualitative Data Analysis: A Methods Sourcebook. 4th edn. Thousand Oaks, CA: Sage Publications; 2020: 61–99.

[45] Hennink MM, Kaiser BN, Marconi VC. Code Saturation Versus Meaning Saturation: How Many Interviews Are Enough? Qual Health Res 2017, 27(4):591–608. 27670770 10.1177/1049732316665344 PMC9359070

[46] Guest G, Bunce A, Johnson L. How Many Interviews Are Enough? Field Methods 2006, 18(1):59–82.

[47] Rural-Urban Continuum Codes [https://www.ers.usda.gov/data-products/rural-urbancontinuum-codes/]

[48] Harris PA, Taylor R, Thielke R, Payne J, Gonzalez N, Conde JG. Research electronic data capture (REDCap)—A metadata-driven methodology and workflow process for providing translational research informatics support. J Biomed Inform 2009, 42(2):377–381. 18929686 10.1016/j.jbi.2008.08.010 PMC2700030

[49] Harris PA, Taylor R, Minor BL, Elliott V, Fernandez M, O’Neal L The REDCap consortium: Building an international community of software platform partners. J Biomed Inform 2019, 95:103208. 31078660 10.1016/j.jbi.2019.103208 PMC7254481

[50] Obeid JS, McGraw CA, Minor BL, Conde JG, Pawluk R, Lin M Procurement of shared data instruments for Research Electronic Data Capture (REDCap). J Biomed Inform 2013, 46(2):259–265. 23149159 10.1016/j.jbi.2012.10.006 PMC3600393

[51] Ridge D, Bullock L, Causer H, Fisher T, Hider S, Kingstone T ‘Imposter participants’ in online qualitative research, a new and increasing threat to data integrity? Health Expect 2023, 26(3):941–944. 36797816 10.1111/hex.13724 PMC10154888

[52] Hill JR, Hoel S, Caldwell C, Zuraw M, Elliott C, Pickett AC Flagged for Fraud: Lessons From 3 Case Studies on Detecting Inauthentic Participants in Online Research. J Med Internet Res 2025, 27:e78554. 41071586 10.2196/78554 PMC12552812

[53] Madeo AC, Bouldin ED, Kaphingst KA, Schlechter CR, Yack M, Hill JL. Prevalence of clinician-ordered genetic testing in rural and urban United States counties: An analysis of the 2022 Health Information National Trends Survey. Prev Med Rep 2025, 57:103163. 40718466 10.1016/j.pmedr.2025.103163 PMC12291549

[54] Zoom. Zoom. In., 6.5.12 edn: Zoom Video Communications; 2025.

[55] Francis JJ, Johnston M, Robertson C, Glidewell L, Entwistle V, Eccles MP What is an adequate sample size? Operationalising data saturation for theory-based interview studies. Psychol Health 2010, 25(10):1229–1245. 20204937 10.1080/08870440903194015

[56] Guest G, Namey E, Chen M. A simple method to assess and report thematic saturation in qualitative research. PLoS One 2020, 15(5):e0232076. 32369511 10.1371/journal.pone.0232076 PMC7200005

[57] Boyatzis RE. Transforming qualitative information: Thematic analysis and code development, 1st edn. Thousand Oaks, CA, USA: Sage Publications; 1998.

[58] Crabtree BF, Miller WL. Template Organizing Style of Analysis. In: Doing Qualitative Research. 3rd edn. Edited by Crabtree BF, Miller WL. Thousand Oaks, CA, USA: Sage Publications; 2023: 215–236.

[59] Microsoft. Microsoft Excel. In., 16.86 edn: Microsoft Corporation; 2024.

[60] Crabtree BF, Miller WL. Immersion/Crystallization Organizing Style of Analysis. In: Doing Qualitative Research. 3rd edn. Thousand Oaks, CA, USA: SAGE Publications; 2023: 237–251.

[61] Braun V, Clarke V. Using thematic analysis in psychology. Qualitative Research in Psychology 2006, 3(2):77–101.

[62] Advanced practice provider [https://www.cancer.gov/publications/dictionaries/cancer-terms/def/advanced-practice-provider]

[63] McCreight MS, Rabin BA, Glasgow RE, Ayele RA, Leonard CA, Gilmartin HM Using the Practical, Robust Implementation and Sustainability Model (PRISM) to qualitatively assess multilevel contextual factors to help plan, implement, evaluate, and disseminate health services programs. Transl Behav Med 2019, 9(6):1002–1011. 31170296 10.1093/tbm/ibz085

[64] Lin GA, Trosman JR, Douglas MP, Weldon CB, Scheuner MT, Kurian A Influence of payer coverage and out-of-pocket costs on ordering of NGS panel tests for hereditary cancer in diverse settings. J Genet Couns 2022, 31(1):130–139. 34231930 10.1002/jgc4.1459 PMC8893352

[65] Wang G, Beattie MS, Ponce NA, Phillips KA. Eligibility criteria in private and public coverage policies for BRCA genetic testing and genetic counseling. Genet Med 2011, 13(12):1045–1050. 21844812 10.1097/GIM.0b013e31822a8113 PMC4537294

[66] Hall MJ, Olopade OI. Disparities in Genetic Testing: Thinking Outside the BRCA Box. J Clin Oncol 2006, 24(14):2197–2203. 16682739 10.1200/JCO.2006.05.5889

[67] Rafi I, Crinson I, Dawes M, Rafi D, Pirmohamed M, Walter FM. The implementation of pharmacogenomics into UK general practice: a qualitative study exploring barriers, challenges and opportunities. Journal of Community Genetics 2020, 11(3):269–277. 32468238 10.1007/s12687-020-00468-2 PMC7295877

[68] Christian-Afflu SN, Sheikh R, Strang A, Chicaiza AM, Oliveros S, Barthelus E Adapting mainstreaming genetic education and clinical communication materials for cancer patients of varying backgrounds. Patient Educ Couns 2026, 146:109489. 41643404 10.1016/j.pec.2026.109489 PMC12959254

[69] White S, Jacobs C, Phillips J. Mainstreaming genetics and genomics: a systematic review of the barriers and facilitators for nurses and physicians in secondary and tertiary care. Genet Med 2020, 22(7):1149–1155. 32313152 10.1038/s41436-020-0785-6

[70] Powell BJ, Waltz TJ, Chinman MJ, Damschroder LJ, Smith JL, Matthieu MM A refined compilation of implementation strategies: results from the Expert Recommendations for Implementing Change (ERIC) project. Implement Sci 2015, 10:21. 25889199 10.1186/s13012-015-0209-1 PMC4328074

[71] Li Y, Howell J, Cimiotti JP. Job preference of nurse practitioners: A simulation approach. Int J Nurs Stud Adv 2025, 9:100406. 40893580 10.1016/j.ijnsa.2025.100406 PMC12395149

[72] Halter M, Wheeler C, Pelone F, Gage H, de Lusignan S, Parle J Contribution of physician assistants/associates to secondary care: a systematic review. BMJ Open 2018, 8(6):e019573. 10.1136/bmjopen-2017-019573 PMC6020983 29921680

[73] Jacobson I, Gully B, Eldien A, McGovern E. Breast cancer screening method prevalences in rural and urban women post-pandemic. The Journal of Rural Health 2025, 41(4):e70089. 41146442 10.1111/jrh.70089

[74] Raspa M, Moultrie R, Toth D, Haque SN. Barriers and Facilitators to Genetic Service Delivery Models: Scoping Review. Interact J Med Res 2021, 10(1):e23523. 33629958 10.2196/23523 PMC7952239

[75] Dusic EJ, Theoryn T, Wang C, Swisher EM, Bowen DJ. Barriers, interventions, and recommendations: Improving the genetic testing landscape. Front Digit Health 2022, 4:961128. 36386046 10.3389/fdgth.2022.961128 PMC9665160

[76] Muessig KR, Zepp JM, Keast E, Shuster EE, Reyes AA, Arnold B Retrospective assessment of barriers and access to genetic services for hereditary cancer syndromes in an integrated health care delivery system. Hered Cancer Clin Pract 2022, 20(1):7. 35144679 10.1186/s13053-022-00213-5 PMC8832647

[77] Yang S, Axilbund JE, OĽeary E, Michalski ST, Evans R, Lincoln SE Underdiagnosis of Hereditary Breast and Ovarian Cancer in Medicare Patients: Genetic Testing Criteria Miss the Mark. Ann Surg Oncol 2018, 25(10):2925–2931. 29998407 10.1245/s10434-018-6621-4

[78] Bélisle-Pipon JC, Vayena E, Green RC, Cohen IG. Genetic testing, insurance discrimination and medical research: what the United States can learn from peer countries. Nat Med 2019, 25(8):1198–1204. 31388181 10.1038/s41591-019-0534-z

[79] Brown-Johnson CG, Safaeinili N, Baratta J, Palaniappan L, Mahoney M, Rosas LG Implementation outcomes of Humanwide: integrated precision health in team-based family practice primary care. BMC Fam Pract 2021, 22(1):28. 33530939 10.1186/s12875-021-01373-4 PMC7856755

[80] Cohen SA, Nixon DM. A collaborative approach to cancer risk assessment services using genetic counselor extenders in a multi-system community hospital. Breast Cancer Res Treat 2016, 159(3):527–534. 27581128 10.1007/s10549-016-3964-z

[81] George A, Riddell D, Seal S, Talukdar S, Mahamdallie S, Ruark E Implementing rapid, robust, cost-effective, patient-centred, routine genetic testing in ovarian cancer patients. Sci Rep 2016, 6:29506. 27406733 10.1038/srep29506 PMC4942815

[82] Illumina. Reimbursement for cystic fibrosis genetic testing in the United States. In. San Diego, CA: Illumina Clinical Diagnostics; 2013.

[83] Shapiro AJ, Kroener L, Quinn MM. Expanded carrier screening for recessively inherited disorders: economic burden and factors in decision-making when one individual in a couple is identified as a carrier. J Assist Reprod Genet 2021, 38(4):957–963. 33501564 10.1007/s10815-021-02084-6 PMC8079588

[84] Chan L, Hart LG, Goodman DC. Geographic access to health care for rural Medicare beneficiaries. J Rural Health 2006, 22(2):140–146. 16606425 10.1111/j.1748-0361.2006.00022.x

[85] State Fact Sheets: United States [https://data.ers.usda.gov/reports.aspx?ID=4035#Pef66bee4f5674160bec0b98012cfca14_2_39iT0]

[86] Bazzi S, Fiszbein M, Gebresilasse M. Frontier Culture: The Roots and Persistence of ‘Rugged Individualism’ in the United States. In.: National Bureau of Economic Research; 2017.

